# Direct differentiation of human pluripotent stem cells into vascular network along with supporting mural cells

**DOI:** 10.1063/5.0155207

**Published:** 2023-08-08

**Authors:** Taylor Bertucci, Shravani Kakarla, Max A. Winkelman, Keith Lane, Katherine Stevens, Steven Lotz, Alexander Grath, Daylon James, Sally Temple, Guohao Dai

**Affiliations:** 1 Neural Stem Cell Institute, Rensselaer, New York 12144, USA; 2 Northeastern University, Department of Bioengineering, Boston, Massachusetts 02115, USA; 3 Weill Cornell Medicine, New York, New York 10065, USA

## Abstract

During embryonic development, endothelial cells (ECs) undergo vasculogenesis to form a primitive plexus and assemble into networks comprised of mural cell-stabilized vessels with molecularly distinct artery and vein signatures. This organized vasculature is established prior to the initiation of blood flow and depends on a sequence of complex signaling events elucidated primarily in animal models, but less studied and understood in humans. Here, we have developed a simple vascular differentiation protocol for human pluripotent stem cells that generates ECs, pericytes, and smooth muscle cells simultaneously. When this protocol is applied in a 3D hydrogel, we demonstrate that it recapitulates the dynamic processes of early human vessel formation, including acquisition of distinct arterial and venous fates, resulting in a vasculogenesis angiogenesis model plexus (VAMP). The VAMP captures the major stages of vasculogenesis, angiogenesis, and vascular network formation and is a simple, rapid, scalable model system for studying early human vascular development *in vitro*.

## I. INTRODUCTION

Human pluripotent stem cell (hPSC)-derived models offer new opportunities to study human vascular development and disease. hPSCs have been successfully differentiated into functional vascular endothelial cells (ECs) using directed 2D differentiation methods.^1^ ECs are isolated from mesodermal derivatives using a general EC surface marker such as CD31 (PECAM) or VE-cadherin (CD144). Isolated ECs have been characterized for important EC functions such as taking up acetylated low-density lipoprotein (LDL), regulating thrombosis and inflammation, forming tubules in angiogenic assays, and responding to flow. Such hPSC-derived ECs have shown promise in a variety of applications such as modeling tissue vascularization and as tissue engineered vascular grafts.^2^ Advanced protocols are emerging to direct differentiation into arterial and venous EC subtypes, several of which manipulate the concentration of vascular endothelial growth factor (VEGF) to bias toward these fates, with low VEGF producing venous ECs and high VEGF levels producing arterial ECs.^3–5^ However, maintaining EC arterial and venous phenotypes in 2D cultures has proved challenging.^6–8^ Phenotypic drift, in which ECs cultured in isolation lose specialized features, impedes *in vitro* studies of arterial and venous ECs. Furthering knowledge of human vascular development and of the signals that govern arterial–venous vessel specification will help advance our ability to maintain and study these cells in tractable culture systems.

During vascular development *in vivo*, ECs first differentiate and self-assemble to form the primitive vascular plexus, in a process known as vasculogenesis. The primitive network is then remodeled through a concert of angiogenic events, in which new vessels sprout from the existing vasculature. During this angiogenic phase, gradients of VEGF induce single ECs to become leading tip cells that sprout into the tissue, guiding vessel growth.^9^ Notch signaling in the tip cells suppresses tip cell features in the immediately adjacent EC, which as a result takes on a stalk cell phenotype. In stalk cells, Notch activation triggers an intracellular cleavage of Notch1 to create the Notch intracellular domain (NICD) that is released from the cell membrane and translocates into the nucleus where it interacts with the transcription factor CSL (CBF1, Suppressor of Hairless, Lag-1) and induces downstream effectors of the stalk cell phenotype.^10^ Concomitant with these two major phases of vasculogenesis and angiogenesis, the blood vessels mature, and key mural support cells, notably pericytes and vascular smooth muscle cells (SMCs), surround the ECs. EC–mural cell interactions play important roles in vessel formation, stabilization, and deposition of the vessel basement membrane.^11,12^ Specification of vascular progenitors (angioblasts) into arterial and venous ECs, which express distinct markers,^13–23^ is also an essential aspect of vessel formation and maturation. In zebrafish, it has been shown that arterial progenitors arise from medial angioblasts in a high VEGF environment, while venous progenitors arise from lateral angioblasts in a low VEGF environment.^24^ This signaling appears to be conserved in humans, as evidenced by human PSC differentiation methods that use different VEGF levels to produce arterial- or venous-biased EC phenotypes.^3–5^ Overall, while the molecular mechanisms involved in these different stages of vascular development have been well established using animal models such as zebrafish and mice,^17,18,21,23,25–30^ they have been less studied in human systems, in large part due to lack of suitable models that recapitulate these events.

Various protocols have been developed to derive ECs,^1^ pericytes,^31,32^ and SMCs^33,34^ from hPSCs. To create 3D vascular models, most prior studies have first derived ECs in 2D cultures, then purified and combined them with mural cells, either with or without a scaffold such as a hydrogel.^35,36^ Others have derived progenitors or co-derived ECs and pericytes in 2D and combined them into a 3D hydrogel.^37,38^ These approaches have the advantage of accurately combining the different vascular cell types in appropriate ratios, but they do not recapitulate the sequential stages of vascular development from the earliest lineage steps. Furthermore, the phenotype of the ECs in these models has not been well characterized (the phenotype varies depending on the EC differentiation protocol used and/or the timing of the model setup), and it is unknown whether they have the capacity to mature into arterial-like and venous-like vessels.

Given the need to study the arc of vascular development from early stages, protocols that generate arterial ECs, venous ECs, and support cell types simultaneously and in 3D offer advantages. For example, recently, co-differentiated 3D hPSC-derived vascular organoids consisting of ECs and pericytes have been described and used to study diabetic vasculopathy.^39,40^ In this case, the identity of the ECs in the 3D construct, whether arterial or venous, was not explored, although after transplantation *in vivo* they differentiated into these two major EC types. This prior work encouraged us to develop a vascular cell co-differentiation protocol, apply it in 3D, and examine whether this 3D model can be used to study early and maturation stages of vascular development and arterial–venous specification in a human cell system.

Here, we report a rapid, serum-free, co-differentiation protocol that works in 2D and 3D to generate ECs, pericytes, and SMCs simultaneously. When this protocol is applied in 2D, ECs can be isolated for 2D culture or the vascular cell mixture can be seeded into a 3D hydrogel system to generate vascular networks. When this protocol is performed in a 3D hydrogel support, the hPSCs directly differentiate into ECs and mural cells that self-organize into a vascular network comprised of arterial-like and venous-like vessel networks supported by juxtaposed mural cells. We show that this 3D model demonstrates the key stages of vasculogenesis, angiogenesis, as well as EC diversification and maturation, showing its utility to study human vascular development, including arterial–venous specification. We term the resulting 3D model a vascular angiogenic model plexus (VAMP). Because VAMPs are produced directly from human PSCs over a 12-day period in a reliable and reproducible manner, production is easily scaled. VAMPs are a rapid, advanced *in vitro* model system to study human vascular development and arterial–venous specification.

## II. RESULTS

### A. 2D vascular co-differentiation protocol generates both mural cells and functional ECs

**FIG. 1.**
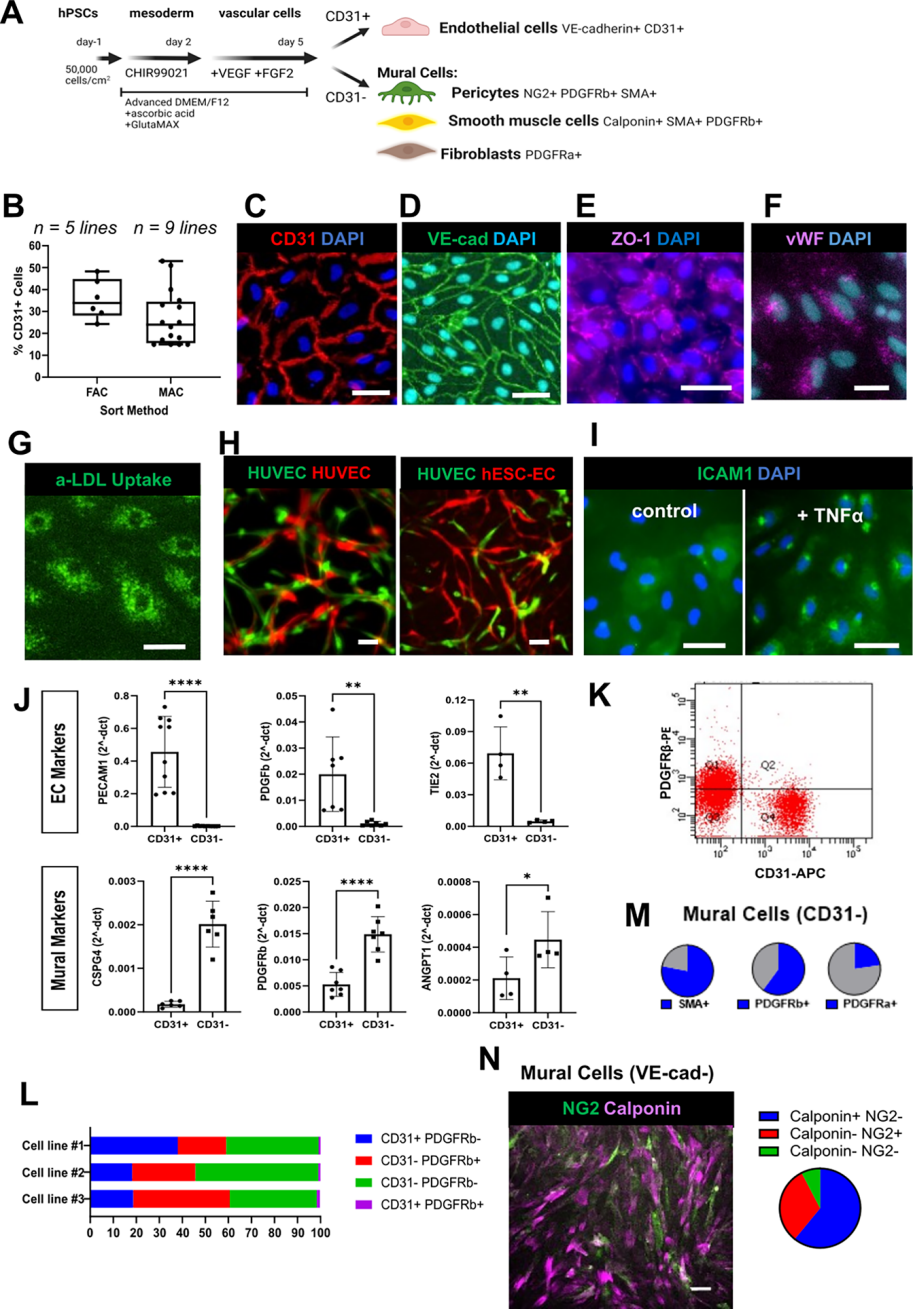
hPSC differentiation in 2D generates mural cells and functional hPSC-ECs. (a) Schematic of the 2D protocol to differentiate hPSCs into vascular cell types. (b) Graph of CD31^+^ cells sorted from five hPSC lines using CD31^+^ FACS and nine hPSC lines using CD31^+^ MACS across n = 6 independent differentiations. (c) Image of hPSC-derived ECs sorted, cultured, and immunostained for the general EC markers CD31 (red) and DAPI (blue). Scale bar = 50 *μ*m. (d) Image of hPSC-derived ECs sorted, cultured, and immunostained for the general EC markers VE-cadherin (green) and DAPI (blue). Scale bar = 50 *μ*m. (e) Image of hPSC-derived ECs sorted, cultured, and immunostained for the tight junction marker ZO-1 (purple) and DAPI (blue). Scale bar = 50 *μ*m. (f) Immunofluorescence image of hPSC-derived ECs sorted, cultured, and immunostained for von Willebrand factor (vWF) (purple) and DAPI (blue). Scale bar = 50 *μ*m. (g) Image of hPSC-derived ECs sorted, cultured, and assayed for LDL uptake. Demonstration of uptake after a 4-hour incubation with 488 tagged acetylated low-density lipoproteins (aLDL). Scale bar = 50 *μ*m. (h) Images of hPSC-derived ECs sorted and HUVECs in a tube forming assay. Fibrin gel seeded with HUVECs (red), HUVECs (green), and fibroblasts (unlabeled) compared to sorted hPSC-derived ECs (red), HUVECs (green), and fibroblasts (unlabeled). Both models were cultured for 7 days and imaged to demonstrate integration of ECs into tube-like structures. Scale bars = 50 *μ*m. (i) Immunofluorescence image of hPSC-derived ECs stained for ICAM1 expression after TNFα treatment (5-h, 50 ng/ml TNFα) compared to untreated hPSC-ECs. Scale bar = 50 *μ*m. (j) Gene expression of EC and mural cell-related genes assessed by qPCR: hPSC-2D-derived CD31^+^ ECs and CD31^−^ mural cells immediately after sorting (2^-dCt^ normalized to GAPDH). Bars represent mean ± standard derivation. n = 4–10 samples, n = 3–6 cell lines. unpaired t-test. ^*^p ≤ 0.1, ^**^p ≤ 0.005, and ^****^p ≤ 0.00005. (k) Representative flow cytometry plot of CD31 (APC-A) and PDGFRβ (PE-A) after 2D hPSC vascular differentiation on day 6. (l) Bar graphs of average percentages of CD31 and PDGFRβ cell populations analyzed on day 6 of differentiation using flow cytometry across three different iPSC lines. n = 2 wells per line. (m) Percent mural cells (CD31-sorted) expressing smooth muscle actin (SMA), PDGFRa and PDGFRb assessed by flow cytometry, n = 6 iPSC lines. (n) Immunofluorescence image (left) and image quantification (right) of the VE-cadherin-negative population sorted on day 6 and cultured for 2 days, then stained for the pericyte marker NG2 (green) and the SMC marker Calponin (purple). Scale bar = 100 *μ*m.

We started with a previously published hPSC serum-free 2D protocol that uses 2 days of WNT activation in advanced DMEM/F12 supplemented with L-glutamine and ascorbic acid (LaSR medium) to stimulate mesoderm differentiation and efficiently produce CD34^+^CD31^+^ vascular progenitor cells capable of differentiating into ECs and SMCs.^41^ Corroborating their finding that inhibition of β-catenin at different times during the first 5 days of the protocol altered the efficiency of EC progenitor production,^41^ we demonstrated that the timing of addition of the WNT activator CHIR99021 was important for generation of the EC fate. Addition of 6 *μ*M CHIR99021 at 48 h maximized the generation of VE-cadherin^+^ ECs within the vascular cell mixture [Fig. S1(a)]. In the original protocol,^41^ after 2 days of WNT activation, the medium was changed to StemPro or LaSR medium for days 2–5, and it was noted that additional VEGF was dispensable for differentiation of CD31^+^CD34^+^ progenitors when combined with StemPro. We found that a combination of 50 ng/ml VEGF and 20 ng/ml fibroblast growth factor 2 (FGF2) added to LaSR medium from days 2–5 effectively generated VE-cadherin^+^ ECs [Fig. S1(b)]. Very few VE-cadherin^+^ ECs were produced when VEGF was omitted or when the VEGF concentration was low (12.5 ng/ml), demonstrating that 20 ng/ml FGF2 during this period was not by itself sufficient to drive EC production in 2D [Fig. S1(b)]. Our refined protocol [Fig. 1(a)] produced approximately 30% ECs, and ECs can be sorted out using magnetic cell sorting (MAC) or fluorescent assisted cell sorting (FAC) methods [Fig. 1(b)]. Importantly, we tested EC generation across 14 iPSC cell lines, each derived from a different donor, and showed the protocol to be fairly consistent in the overall ability to drive EC differentiation as well as individual variability of % ECs generated across these cell lines [Fig. 1(b)].

The original protocol^18^ demonstrated that day 5-sorted vascular progenitors were capable of differentiation into ECs expressing characteristic EC markers VE-cadherin, von Willebrand factor (vWF), and intercellular adhesion molecule 1 (ICAM1) after a further 15 days in EGM-2 medium (PromoCell). In our experiments, we sorted ECs between days 5 and 8 using either VE-cadherin or CD31 as a positive selection marker. The selected hPSC-ECs were then cultured for 0–1 passages (2–8 days), and they formed confluent monolayers that robustly expressed CD31, VE-cadherin, vWF, and ZO-1, another distinctive EC marker [Figs. 1(c)–1(f)]. This sorted population of hPSC-ECs also showed important functions of ECs: they demonstrated acetylated LDL uptake [Fig. 1(g)], integrated with human umbilical vein ECs (HUVECs) to form tube-like structures [Fig. 1(h)] and responded to the inflammatory cytokine TNFα via upregulation of ICAM1 [Fig. 1(i)]. We found the trans-endothelial electrical resistance (TEER) across the confluent hPSC-EC monolayers to be approximately 40 Ω cm^2^, which is in a similar range to cultured HUVECs.^42^ In addition to ECs, this protocol also generates perivascular support cells, also known as mural cells. CD31-positive and CD31-negative sorted cell populations are consistent with EC and mural cell marker profiles, respectively. CD31^+^ cells measured higher expression of EC-related genes, *PECAM1*, *PDGFβ*, and *TIE2* (receptor for angiopoetin-1), and CD31^−^ cells expressed higher levels of mural cell markers: *CSPG4* (gene that encodes for NG2), *PDGFRβ* (gene that codes for PDGFβ receptor), and *ANGPT1* (gene that codes for angiopoietin-1, ligand for TIE2) [Fig. 1(j)].

From the original protocol, it was demonstrated that day 5-sorted vascular progenitors were capable of generating mural cells illustrated by Calponin-1^+^ SMCs after 10 days in SmGM-2 (Lonza).^18^ In our protocol, we quantified that approximately half of the CD31^−^ cells were positive for pericyte marker PDGFRβ on day 6 of differentiation—this was consistent across hPSC cell lines [Figs. 1(k) and 1(l)]. CD31^−^ cells sorted on day 5 were also positive for smooth muscle actin (SMA) and fibroblast marker PDGFRα [Fig. 1(m)]. We also demonstrated that this population, sorted on day 6 and cultured for 2 additional days, is comprised of cells that stain positive for SMC marker Calponin-1 and pericyte marker NG2 [Fig. 1(n)]. Taken together, these properties confirm that the protocol we have developed produces both a functional EC population and two major classes of mural cells.

### B. 2D vascular differentiation protocol generates a mixture of ECs and mural cells that create vascular networks when seeded into 3D hydrogel and perfusable vascular networks when seeded into a microfluidic device

**FIG. 2.**
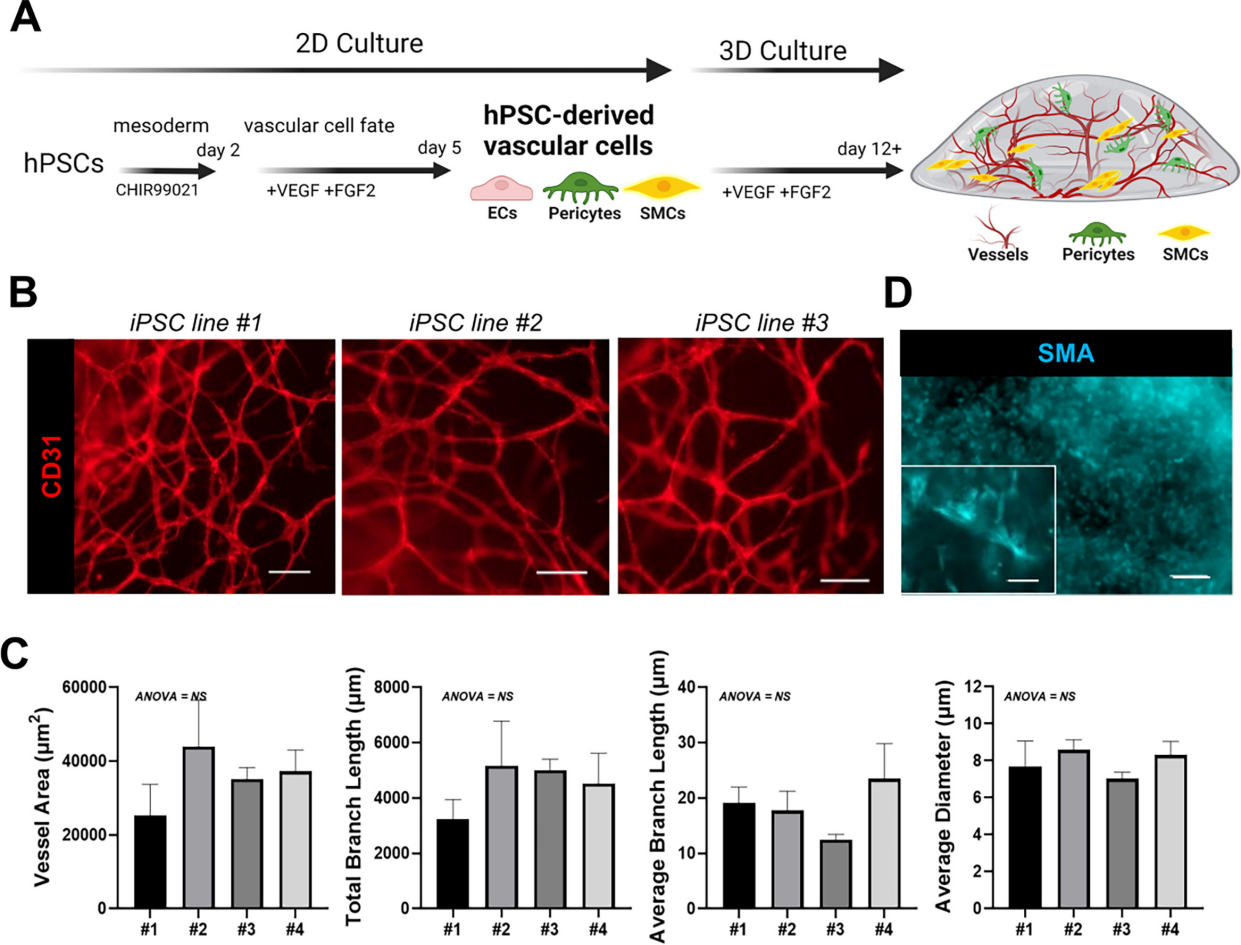
hPSC vascular cells differentiated in 2D and then seeded into a 3D hydrogel create vascular networks. (a) Schematic of the protocol for generating a 3D model by differentiating hPSCs into vascular cells in 2D and then remixing and seeding the cells in a hydrogel support to form 3D vascular networks. (b) Immunofluorescence representative images of hPSC vascular models from three different iPSCs after 25 days of 3D culture in fibrin hydrogel stained for CD31 (red). Scale bar = 100 *μ*m. (c) Vascular network quantification of CD31 immunofluorescence images across four model replicates for vessel area, total branch length, average branch length, and average vessel diameter. n = 4 models across 2 iPSC lines. Bars represent mean ± standard deviation. One-way ANOVA, NS = not significant. (d) Immunofluorescence image of hPSC vascular models after 7 days of 3D culture stained with smooth muscle actin to label mural cells. Scale bar = 200 *μ*m; inset scale bar = 50 *μ*m.

In order to determine if the 2D PSC-derived vascular cells can be used to create a 3D vasculature model, we seeded the unsorted mixture comprised of ECs and mural cells into a 3D scaffold. After the 6-day differentiation protocol, cells were lifted from the 2D culture by enzymatic dissociation and directly reseeded into a 3D hydrogel [Fig. 2(a)]. Over 7 days, ECs undergo vascular tube formation, structures that remain stable for at least 25 days of 3D culture and show consistency in vessel structures between replicates and cell lines [Figs. 2(b) and 2(c)]. Mural cells positive for SMA were observed throughout the 3D construct [Fig. 2(d)].

**FIG. 3.**
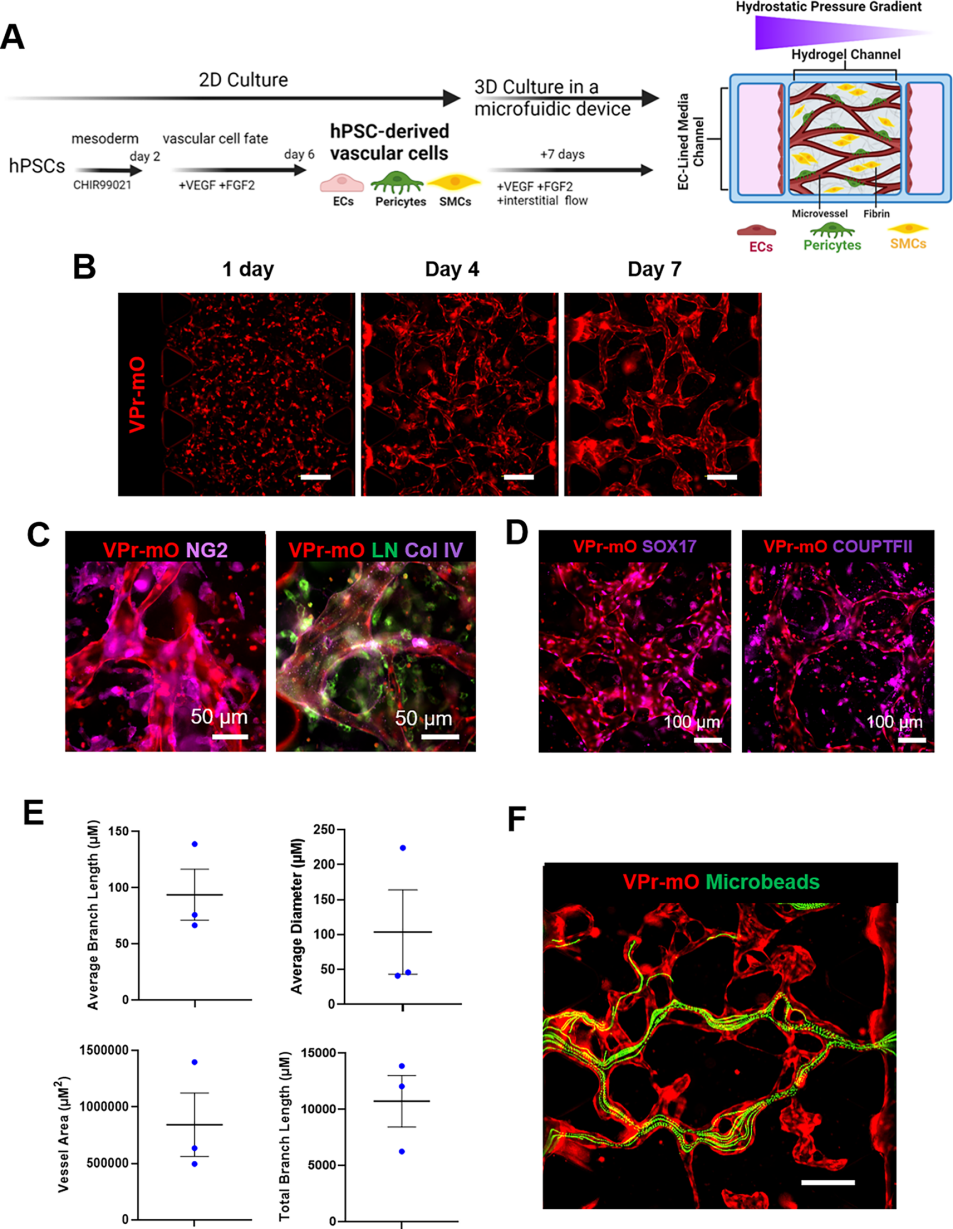
hPSC vascular differentiation in 2D seeded into a microfluidic device creates perfusable vascular networks. (a) Schematic of the protocol for generating a 3D model by differentiating hPSCs into vascular cells in 2D and then remixing and seeding the cells in a hydrogel channel within a microfluidic device. The cross-sectional view illustrates the hydrostatic pressure gradient established across the 3D vascular model to promote open lumen vessels. (b) Images of the hPSC-derived ECs over time within the microfluidic device. Images taken days 1, 4, and 7 after seeding into the microfluidic device. ECs were visualized using the VPr-mOrange reporter. Scale bars = 100 *μ*m. (c) Immunofluorescence images of open lumen vessels stained for pericytes (NG2) and basement membrane proteins (laminin, collagen IV) after 7 days inside the microfluidic device (day 13 of differentiation). ECs were visualized using the VPr-mOrange reporter. Scale bars = 50 *μ*m. (d) Immunofluorescence images of open lumen vessels stained for arterial marker SOX17 and venous marker COUPTFII after 7 days inside the microfluidic device (day 13 of differentiation). ECs were visualized using the VPr-mOrange reporter. Scale bars = 100 *μ*m. (e) Vascular network quantification across three microfluidic device model replicates for vessel area, total branch length, average branch length, and average vessel diameter. ECs were visualized using the VPr-mOrange reporter. Bars represent mean ± standard deviation. (f) Maximum projection image of 488-tagged microbeads (green) traveling through open lumen vessels in the microfluidic device after 7 days of culture within the microfluidic device. ECs visualized using the VPr-mOrange reporter. Scale bar = 100 *μ*m.

Additionally, we introduced the unsorted mixture of vascular cells into a hydrogel within a microfluidic device (AIM Biotech) and set up a hydrostatic pressure difference between opposite reservoirs of the device to induce interstitial flow across the hydrogel channel [Fig. 3(a)]. Bulk fluid movement through the hydrogel promoted the formation of vessel structures and anastomosis of the developing microvessels. We observed hPSC-ECs organizing into tube structures over time in the microfluidic device [Fig. 3(b)]. Immunostaining of the cells in the device demonstrated that pericytes, identified by the surface marker NG2,^43^ were localized near the vessels, and that there was basement membrane deposition (laminin and collagen IV), [Fig. 3(c)], as expected, with endothelial–pericyte interactions.^11,12^ Certain vessels were observed to be positive for arterial marker SOX17 or venous marker COUP-TFII [Fig. 3(d)]. Vascular structures were analyzed and found to be similar across replicates [Fig. 3(e)]. To confirm anastomosis of open lumen tubes, we performed a fluorescent microsphere perfusion assay. After 7 days of culture within the microfluidic device, fluorescent microspheres were added to one channel of the microfluidic device and a differential pressure was applied to allow beads to travel across the device. We demonstrated anastomosis and open vessels as microbeads were observed to move across the device through the model vasculature [Fig. 3(f), supplementary material, Video 1].

In summary, we have developed a straightforward method for creating hPSC-derived 3D vascular models containing open lumen vessels within 2 weeks.

### C. 2D vascular differentiation protocol generates arterial-like ECs, but arterial specification is lost in 2D culture

**FIG. 4.**
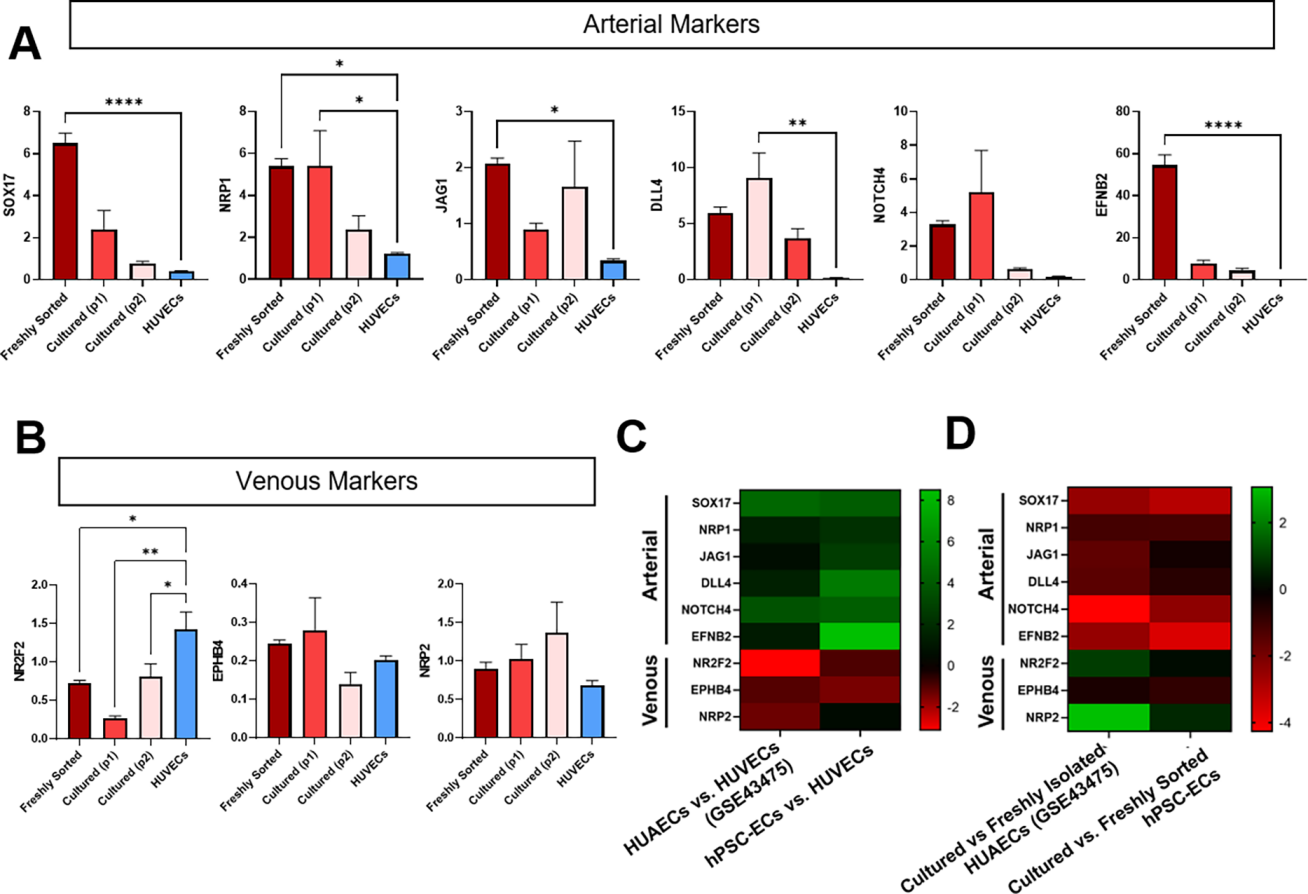
hPSC vascular differentiation in 2D generates functional hPSC-ECs with an arterial-like phenotype. (a) Gene expression of arterial-related genes assessed by qPCR: hPSC-2D-derived ECs immediately after sorting (freshly sorted), cultured for 2–4 days for one (p1) or 7–9 days for two passages (p2) and HUVECs (p4) (2^-dCt^^*^10^5^ normalized to Rn18s). Bars represent mean ± standard error. n = 3 samples, ANOVA, post hoc compared to HUVECs. ^*^p ≤ 0.05, ^**^p ≤ 0.005, and ^****^p ≤ 0.00005. (b) Gene expression of venous-related genes assessed by qPCR: hPSC-2D-derived ECs immediately after sorting (freshly sorted), cultured for 2–4 days for one (p1) or 7–9 days for two passages (p2) and HUVECs (p4) (2^-dCt^^*^10^5^ normalized to Rn18s). Bars represent mean ± standard error. n = 3 samples, ANOVA, post hoc compared to HUVECs. ^*^p ≤ 0.05 and ^**^p ≤ 0.005. (C) Heat map of gene expression (logFC) of arterial- and venous-related genes. Column one compares freshly isolated HUAECs to HUVECs from dataset GSE43475. Green indicates higher gene expression in HUAECs compared to HUVECs. Red indicates lower gene expression in HUAECs compared to HUVECs. Column two reports logFC from our qPCR dataset between immediately after sorting hPSC-ECs (freshly sorted) to HUVECs (p4). Green indicates higher gene expression in hPSC-ECs compared to HUVECs. Red indicates lower gene expression in hPSC-ECs compared to HUVECs. (d) Heat map of gene expression (logFC) of arterial and venous related genes. Column one compares cultured to freshly isolated HUAECs from dataset GSE43475. Green indicates higher gene expression in cultured compared to freshly isolated HUAECs. Red indicates lower gene expression in cultured compared to freshly isolated HUAECs. Column two reports logFC from our qPCR dataset compares cultured (p2) to immediately after sorting hPSC-ECs (freshly sorted). Green indicates higher gene expression in cultured compared to freshly sorted hPSC-ECs. Red indicates lower gene expression in cultured compared to freshly sorted hPSC-ECs.

To examine the arterial–venous EC identity of the ECs derived from this 2D vascular differentiation protocol, we compared the gene expression profiles of hPSC-derived ECs and HUVECs. hPSC-ECs expressed significantly higher levels of several arterial-associated genes, including *SOX17, NRP1, JAG1, DLL4,* and *EFNB2*, and lower levels of the vein-related genes, *EPHB4 and NR2F2*, compared to cultured HUVECs [Figs. 4(a) and 4(b)]. We then compared our data to transcriptomic data previously obtained from freshly isolated human umbilical arterial ECs (HUAECs) and HUVECs.^6^ In that study, a set of genes was identified that were differentially expressed in HUAECs compared to HUVECs. The arterial-associated genes we identified as higher in hPSC-derived ECs compared to HUVECs were also higher in HUAECs compared to HUVECs in that study [Fig. 4(c)]. The venous-associated genes we identified as lower in hPSC-derived ECs compared to HUVECs were also lower in HUAECs compared to HUVECs in that study [Fig. 4(c)]. Together, these data suggest that ECs differentiated from this 2D protocol are initially biased toward an arterial fate.

We found that this bias toward arterial identity was lost over time in 2D culture. In particular, the arterial-associated genes *SOX17, NRP1, NOTCH4*, and *EFNB2* were significantly downregulated after two passages [Fig. 4(a)]. This was not unexpected as prior studies have shown that primary isolated ECs demonstrate phenotypic drift such as loss of tissue-specific or blood vessel type-specific markers, when cultured for 2–15 days in 2D.^6–8,44^ We compared our data to transcriptomic data previously obtained from freshly isolated HUAECs and HUAECs cultured for 2–3 passages (10–15 days), which identified genes significantly downregulated in cultured HUAECs.^6^ The arterial-associated genes that were downregulated in cultured hPSC-derived ECs were also downregulated when HUAECs were cultured in that study [Fig. 4(d)].

The VEGF concentration had a large impact on the efficacy of EC differentiation in 2D [Fig. S1(b)], and it impacted expression of several genes associated with arterial–venous specification (Fig. S2). The arterial-associated Notch ligand genes *JAG1* and *DLL4* were downregulated, and the venous marker *NR2F2* was upregulated when ECs were derived with low VEGF (12.5 ng/ml) compared to high VEGF (50 ng/ml). However, the arterial-related genes *SOX17, NRP1, NOTCH4,* and *EFNB2* and the venous-related gene *EPHB4* were not significantly changed. Overall, VEGF concentration is able to influence the expression of a few characteristic arterial–venous related genes.

Hence, this 2D protocol generates ECs that are functional, but with time in culture, over a period of 1–2 passages, they lose arterial–venous identity, demonstrating the phenotypic drift previously reported for 2D cultures of purified ECs directly isolated from tissues or generated from hPSCs.^45^ Note that the length of time ECs have been cultured is an important variable to consider when creating a 3D remixed cell model. Moreover, when vascular cells are generated in 2D and then introduced into a 3D model by remixing, the early events of vascular development are missed. This led us to translate the 2D protocol into a 3D differentiation protocol with two main goals: first to recapitulate early vascular development events and second to determine if this approach would achieve a more stable arterial–venous EC phenotypic commitment.

### D. VAMPs exhibit vasculogenic, angiogenic events and vessel maturation: EC–mural cell interactions and basement membrane deposition

**FIG. 5.**
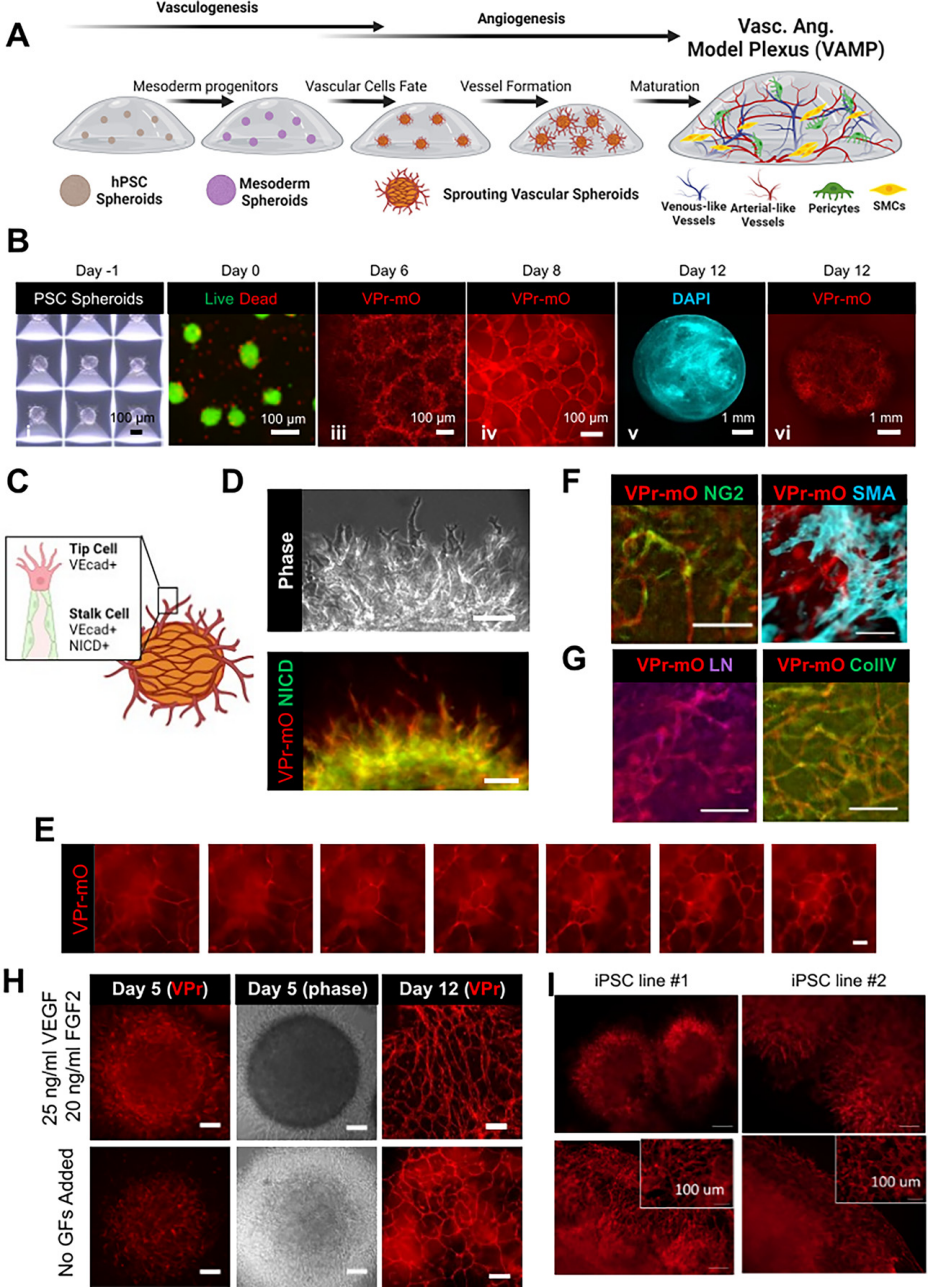
VAMPs demonstrate vasculogenic, angiogenic, and vessel maturation events. VAMPs can be biased toward angiogenic events when VEGF and FGF2 are added into the medium. (a) Schematic showing the development of the VAMP model. hPSC spheroids are encapsulated into a hydrogel support and differentiated into ECs, pericytes, and smooth muscle cells that self-assemble into vascular networks via vasculogenic and angiogenic behaviors. (b) (i) Phase image of hPSC spheroids generated in an Aggrewell dish on day 1. Scale bar = 100 *μ*m. (ii) Image of live (calcein AM) and dead (ethidium homodimer-1) cells after spheroid encapsulated into a fibrin hydrogel on day 0. Scale bars = 200 *μ*m. (iii) Image of ECs visualized by VPr-mOrange reporter within and sprouting from spheroids within a fibrin hydrogel on day 6. Scale bar = 100 *μ*m. (iv) Image of EC networks visualized by VPr-mOrange reporter assembled within a fibrin hydrogel on day 8. Scale bar = 100 *μ*m. (v) Low-power image of VAMP construct stained with DAPI on day 12. Scale bar = 1 mm. (vi) Low-power image of VAMP construct of ECs visualized by VPr-mOrange reporter on day 12. Scale bar = 1 mm. (c) Schematic of tip and stalk cell phenotypes sprouting from vascular spheroids encapsulated in a hydrogel. (d) Image of cells sprouting from spheroids encapsulated in a fibrin gel on day 5. ECs were visualized with the VE-cadherin-promotor-mOrange reporter (VPr-mO) and immunostained for the NOTCH1 intracellular domain (NICD). Scale bars = 100 *μ*m. (e) Z-stack images through a VAMP construct on day 12 (∼800 *μ*m thick). Vascular structures were visualized with the VPr-mOrange reporter. Scale bar = 100 *μ*m. See the supplementary material, Video 4. (f) Immunofluorescence of VAMPs at day 12. Vascular structures were visualized with the VPr-mOrange reporter and stained for mural cells: pericytes (NG2) and SMCs [smooth muscle actin (SMA)]. Scale bars = 100 *μ*m. (g) Immunofluorescence of VAMPs at day 12. Vascular structures were visualized with the VPr-mOrange reporter and stained for basement membrane protein deposition: collagen IV (Col IV) and laminin (LN). Scale bars = 50 *μ*m. (h) Images of 3D vascular models on day 5 and day 12. Models were generated from 3D differentiation protocols by adding 25 ng/ml VEGF and 20 ng/ml FGF2 into the medium on day 2 compared to no growth factors added. ECs were visualized using with the VPr-mOrange reporter. Scale bars = 100 *μ*m. (i) Images of VAMP models derived from additional iPSC lines. Images taken on day 6 (top images) demonstrate sprouting behavior and images taken on day 14 (bottom images) demonstrate vascular network formation. Scale bars = 200 *μ*m. Inset scale bar = 100 *μ*m.

Our goal was to create a 3D EC and mural cell co-differentiation protocol that mimics key events of vascular development. To enable real-time observation of vascular differentiation and network formation during this process, we used an hESC line (WMC-2 hESCs) carrying a VE-cadherin-promoter-mOrange reporter (VPr-mO)^46^ that fluoresces when cells differentiate into ECs. First, the WMC-2 hESCs were plated into AggreWell plates to form spheroids with diameters of ∼100 μm [Fig. 5(b)]. After 1 day, the spheroids were removed from the wells, and multiple spheroids (∼10–20) were encapsulated into a single fibrin hydrogel. The sequence of medium changes developed in the 2D protocol was then performed on the 3D-encapsulated spheroids [Figs. 5(a) and 5(b)].

We found that the spheroids exhibited developmental steps characteristic of vascular development. The cells first self-assembled into a primitive 3D vascular network. Starting at day 4, a network of cells expressing VPr-mO were found within particular spheroids, while others sprouted extending their networks into the surrounding hydrogel matrix (supplementary material, Videos 2 and 3). Sprouting cells demonstrated tip cell morphology at the leading edge, while cells positioned at the base of these sprouts expressed NICD, consistent with the EC tip and stalk cell phenotypes observed in angiogenesis [Figs. 5(c) and 5(d)]. Between days 8 and 12, the vascular sprouting spheroids had expanded and remodeled the hydrogel support, resulting in a cell-dense construct comprised of interconnected vascular networks [Figs. 5(b) and 5(e), supplementary material, Video 4]. At this stage, we evaluated the interaction between ECs, pericytes, and SMCs within the 3D construct. NG2-expressing pericytes were localized around vessel structures, while SMCs, identified by expression of smooth muscle actin (SMA) were observed throughout the construct [Fig. 5(f), supplementary material, Video 5]. We observed basement membrane proteins deposited around vessel structures [Fig. 5(g)]. We were also capable of applying this protocol to generate vascular networks within a microfluidic device (Fig. S3). Given that the model exhibits signs of vasculogenesis, angiogenesis, and formed a plexus-like network, we termed this 3D model VAMP.

### E. Exogenous VEGF and FGF2 are not required for vascular network formation but enhance angiogenic sprouting in VAMPs

We demonstrated the essential role of exogenous VEGF in the 2D differentiation protocol [Fig. S1(b)], but we found this was not essential for generation of 3D VAMPs. Vascular networks were robustly generated with or without added VEGF and FGF2 in the medium. It is likely that in a VAMP, the endogenous growth factors produced by the cells are sufficient to drive vascular differentiation. Smooth muscle cell-derived VEGF production has been reported to drive network formation and maturation in 2D co-cultures of primary SMCs and HUVECs.^47^ In contrast, VEGF and FGF2 medium supplements, a synergistic pro-angiogenic combination,^48^ did impact angiogenic events within the VAMP. When VEGF and FGF2 were added, the number of ECs sprouting outward into the matrix noticeably increased, consistent with studies that demonstrate sprouting behaviors can be stimulated by VEGF or VEGF combined with FGF2 in primary ECs^49,50^ and in PSC-derived ECs.^35,51^ In contrast, in VAMPs derived without added VEGF and FGF2, EC networks were initially localized within the vascular spheroid centers and much less outward sprouting was observed [Fig. 5(h), supplementary material, Videos 6 and 7]. VAMPs derived with or without added VEGF and FGF2 successfully generated vascular networks, albeit with slightly different morphologies [Fig. 5(h)]. Hence, we are able to manipulate angiogenic behaviors within the 3D VAMP model. VAMP models showed consistent timing and production of vascular structures across iPSC [Fig. 5(i)].

### F. ECs generated in VAMPs express arterial and venous markers

**FIG. 6.**
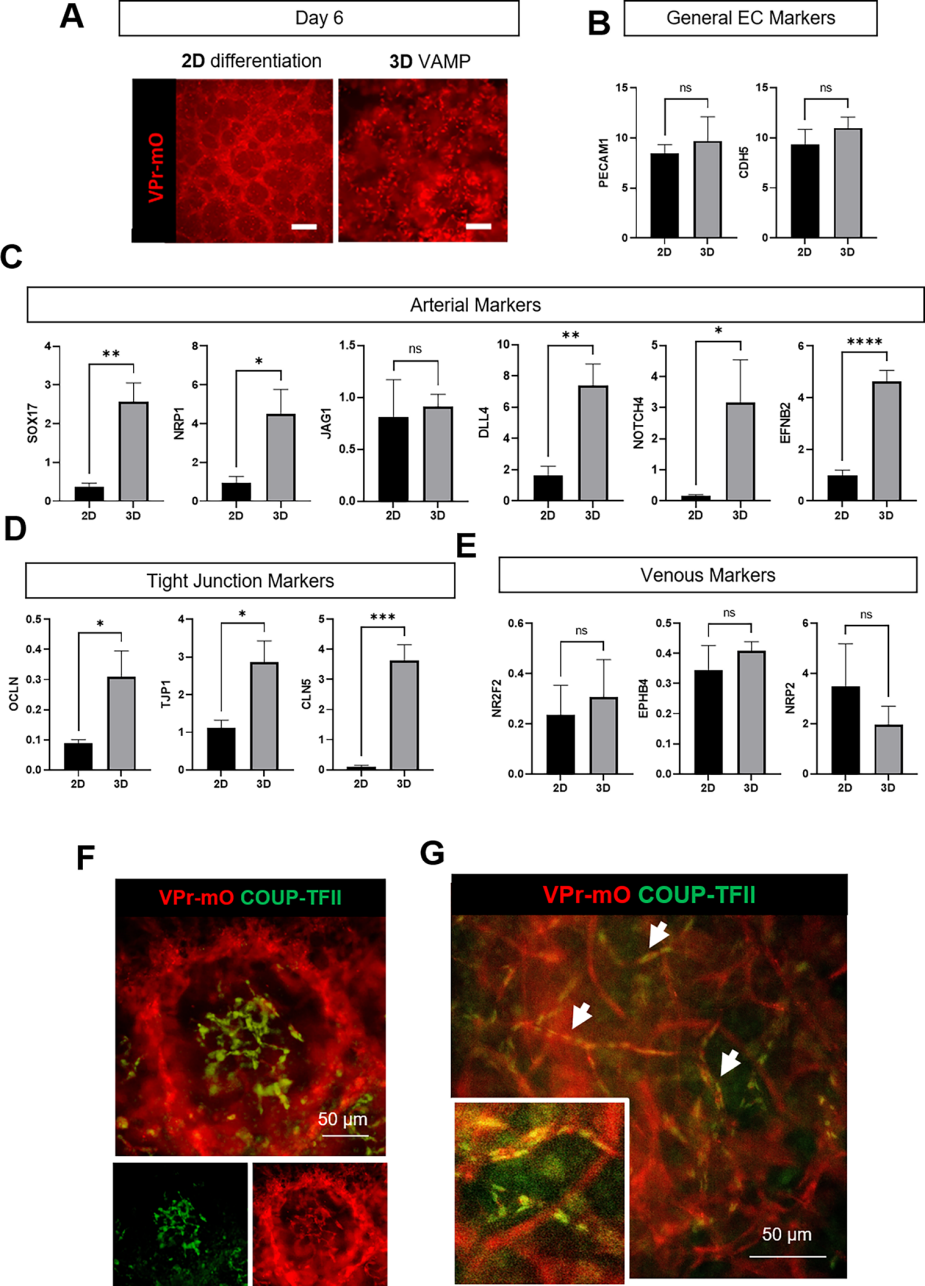
VAMPs generate vascular networks with evidence of early arterial–venous specification. (a) Images of 2D differentiation and 3D VAMP on day 6 prior to EC isolation. 2D differentiation and 3D VAMPs were both generated with 50 ng/ml VEGF and 20 ng/ml FGF2. ECs were visualized using with the VPr-mOrange (VPr-mO) reporter. Scale bars = 100 *μ*m. (b) Gene expression comparison of 3D VAMP-derived ECs to 2D-derived ECs for general endothelial genes via qPCR (2^-dCt^^*^10^5^ normalized to Rn18s) on day 6. Bars represent mean ± standard error. n ≥ 3, unpaired t-test. NS = not significant. (c) Gene expression comparison of 3D VAMP-derived ECs to 2D-derived ECs for arterial-related genes via qPCR (2^-dCt^^*^10^5^ normalized to Rn18s) on day 6. Bars represent mean ± standard error. n ≥ 3, unpaired t-test. ^*^p ≤ 0.05, ^**^p ≤ 0.005, and ^****^p ≤ 0.000 05. (d) Gene expression comparison of 3D VAMP-derived ECs to 2D-derived ECs for tight junction-related genes via qPCR (2^-dCt^^*^10^5^ normalized to Rn18s) on day 6. Bars represent mean ± standard error. n ≥ 3, unpaired t-test. ^*^p ≤ 0.05 and ^***^p ≤ 0.0005. (e) Gene expression comparison of 3D VAMP-derived-ECs to 2D-derived ECs for venous-related genes via qPCR (2^-dCt^^*^10^5^ normalized to Rn18s) on day 6. Bars represent mean ± standard error. n ≥ 3, unpaired t-test. NS = not significant. (f) Immunofluorescence of VAMPs on day 6. VAMPs were stained for the venous marker COUP-TFII, and ECs were visualized using the VPr-mOrange reporter. ECs are observed both sprouting from the spheroid and within the spheroid. COUP-TFII stains localized within spheroid boundary. Scale bars = 100 *μ*m. (g) Immunofluorescence of VAMPs on day 12. VAMPs were stained for the venous marker COUP-TFII, and vascular structures were visualized using the VPr-mOrange reporter. Arrows indicate vessels that are COUP-TFII-positive. Scale bar = 50 *μ*m.

When translating the vascular differentiation protocol from 2D to 3D, we observed several differences. First, ECs were generated more quickly in 3D; ECs were present starting at day four in 3D compared to day five in 2D. We found the expression levels of several genes were also different following 2D and 3D production methods. For this comparison, VE-cadherin^+^ cells were isolated from the total vascular cell mixture from 2D or 3D cultures. For 3D, we chose day 6 to isolate cells because this timepoint was after VE-cadherin^+^ ECs were seen in the VAMP but before full networks were established; to enable a direct comparison, we also selected day 6 for 2D cultures [Fig. 6(a)]. 2D or 3D culture did not significantly affect expression of general EC genes [Fig. 6(b)]. However, ECs derived in 3D expressed significantly higher level of arterial-associated genes (*SOX17, NRP1, DLL4, NOTCH4,* and *EFBN2*) and tight junction genes (*OCLN, TJP1,* and *CLDN5*) compared to 2D ECs [Figs. 6(c) and 6(d)]. In contrast, no changes in venous-related genes *NR2F2, EPHB4,* and *NRP2* were detected when comparing the 3D vs 2D protocols [Fig. 6(e)].

The venous phenotype requires active transcriptional regulation by the *NR2F2* gene product, the COUP-TFII protein.^23^ We identified a sub-population of ECs in 3D spheroids on day 6 that strongly expressed COUP-TFII protein by immunostaining [Fig. 6(f)], consistent with these being venous-like ECs, but this sub-population was not observed in cultures derived in 2D. Note that we did not see any significant difference in expression of *NR2F2* in 2D compared to 3D cultures, so this difference in COUP-TFII protein expression may reflect a difference in translation or the NR2F2 expression is only in a subset of the cells in 3D and so the overall expression does not differ much from the 2D condition. Most interestingly, as VAMPs matured to day 12, they demonstrated the formation of two diverging EC vessel networks [Fig. 6(g)]: vessels comprised of ECs that express COUP-TFII indictive of a venous-like phenotype and vessels that were negative for COUP-TFII, suggestive of a more arterial-like commitment, confirmed by gene expression described above. These events indicate VAMPs can recapitulate early developmental arterial–venous specification.

Taken together, our data suggest that differentiating hPSCs into vascular cells in a 3D environment is important to generate arterial–venous diverging EC phenotypes. This specification has not been shown previously when ECs were derived in 2D and then remixed and cultured in a 3D hydrogel.

### G. VAMPs show differential responses to vascular developmental cues

To illustrate the utility of VAMPs as a tool for studying vascular development, we screened various developmental pathway perturbations for impacts on EC differentiation and vessel formation. We chose signaling pathways for WNT, Notch, sonic hedgehog (SHH), and bone morphogenic protein (BMP) based on their known roles in vascular development, knowledge gained largely from animal models.

**FIG. 7.**
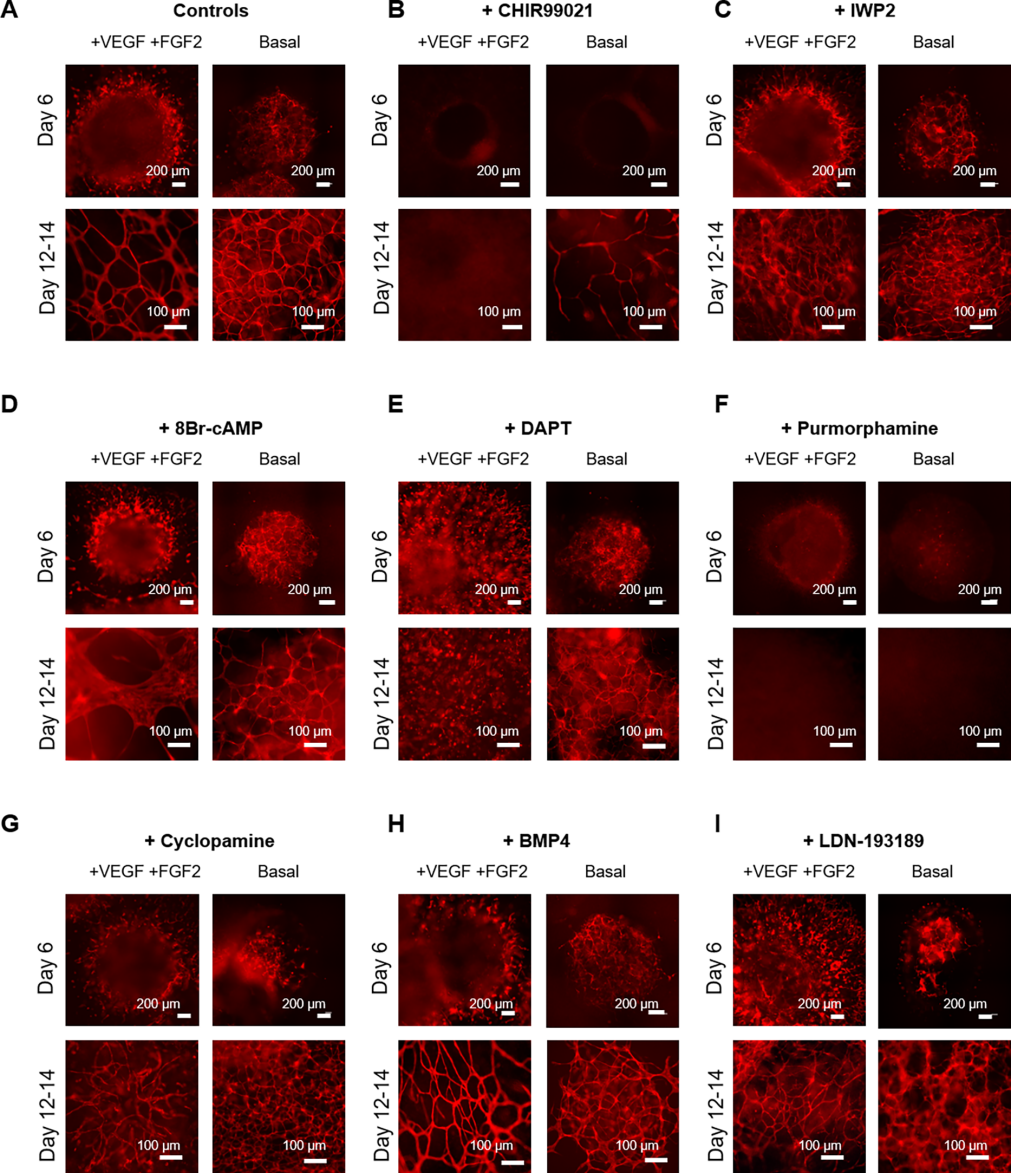
VAMPs have utility as a screening tool for vascular development. (a)–(i) Representative images of VAMPs generated with 50 ng/ml VEGF and 20 ng/ml FGF2 added into the medium from days 2 to 12 compared to no added VEGF and FGF2 (Basal) and treated with agonists or inhibitors between days 2 and 12. Treatments include 6 *μ*M CHIR99021 (b), 5 *μ*M IWP2 (c), 100 *μ*M 8Br-cAMP (d), 20 *μ*M DAPT (e), 2 *μ*M purmorphamine (f), 2 *μ*M cyclopamine (g), 20 ng/ml BMP4 (h), and 250 nM LDN-193189 (i). ECs were visualized using the VPr-mOrange reporter imaged on day 6 and between days 12 and 14.

During this screen, VAMPs were generated under pro-angiogenic conditions (VEGF and FGF2 added between days 2 and 12) and basal conditions (no VEGF or FGF2 added) as controls. For test conditions, we added various agonists/inhibitors of these selected pathways between days 2 and 12. Endothelial differentiation and vessel morphogenesis were monitored over time, and at the 2-week timepoint, vascular structures were analyzed (Figs. 7 and S5). VAMPs generated with VEGF and FGF2 had increased EC sprouting at early timepoints, as also seen in Fig. 5(h). However, the resulting vascular structures displayed no significant differences in vessel diameters, branch length, and number of branches compared to basal conditions. We measured an approximately 10% increase in total vessel area in VAMPS derived in basal condition compared to VAMPs generated with pro-angiogenic conditions [Figs. 7(a) and S5(a)].

The WNT/β-catenin pathway has been implicated in angiogenesis and vascular cell differentiation and recently has been linked to SOX17 expression in arterial ECs.^52–55^ To understand how WNT activation would affect VAMPs after mesoderm commitment, we added CHIR99021 between days 2 and 12. We observed no VPr-mO-positive ECs at any timepoint in VEGF and FGF2 supplemented constructs and very few in basal conditions [Fig. 7(b)]. We noted that the few structures (less than 5% vessel area) formed in basal conditions with CHIR99021 with thinner vessels of similar branch lengths to control samples [Fig. S5(b)].

Conversely, treatment with the WNT production inhibitor IWP2 between days 2 and 12 produced vascular networks when added to VEGF and FGF2 or basal conditions [Fig. 7(c)], with similar percent of vessel area, branch length, and vessel diameters to control samples [Fig. S5(b)]. When IWP2 was added with VEGF and FGF2, vessels exhibited longer branches and larger diameters compared to VAMPs generated when IWP2 was added to basal medium alone. These data demonstrated an interaction between WNT, VEGF, and FGF2 pathways in VAMP models.

Cyclic adenosine monophosphate (cAMP) is a ubiquitous second messenger that can modulate metabolism, cell proliferation, and differentiation. The cAMP analog 8Br-cAMP has been used to increase the efficiency of EC generation and decrease the generation of non-EC cell types from human PSCs.^45^ In VAMPs, the addition of 8Br-cAMP together with VEGF and FGF2 between days 2 and 12 led to more EC differentiation but negatively impacted vessel formation, as demonstrated by production of large EC-sheet like structures [Fig. 7(d)]. Interestingly, addition to basal conditions did not yield the same result, and vessels were generated similar to basal control VAMPs [Fig. 7(d)]. No detectable differences were measured in vessel area, vessel branch length, or vessel diameter [Fig. S5(b)]. EC-sheet like structures were not considered vessels and were omitted from the analysis. These data suggest interaction between 8Br-cAMP, VEGF, and FGF2 in impairing vessel formation.

The Notch pathway is involved in many aspects of vascular development: arterial–venous fate, cell differentiation, proliferation, and angiogenesis.^56–61^ Consistent with this, when the small molecule Notch inhibitor DAPT was added together with VEGF and FGF2 during days 2–12 of the protocol, we failed to observe cells with angiogenic EC morphologies [Fig. 7(e)]. Many ECs were still generated throughout the construct, but they had a rounded morphology, and evidence of vessel structures was lacking. However, unexpectedly, when DAPT was added to VAMPs generated in the basal condition (without added VEGF and FGF2), this was not the case: vascular networks were still generated with no significant change in vessel area, albeit with thinner vessels and shorter branch lengths compared to control [Fig. S5(b)]. Hence, we show using the VAMP model that there is an interaction between DAPT, VEGF, and FGF2 in a vascular structure formation.

SHH signals, originating from the primitive endoderm, are believed to be pro-angiogenic and important for arterial fates^62^ and based on these observations in animal models, we expected treatment with the SHH agonist purmorphamine to be pro-angiogenic. Surprisingly, adding purmorphamine between days 2 and 12 significantly limited the EC fate in both VEGF and FGF2 supplemented and basal conditions [Fig. 7(f)]: no vessel structures were detected.

On the other hand, VAMPs treated with the SHH inhibitor cyclopamine between days 2 and 12 did form vascular networks [Fig. 7(g)], and no detectable differences in vessel structures were measured compared to control samples [Fig. S5(b)]. Interestingly, VAMPs produced with cyclopamine treatments combined with VEGF and FGF2 produced vessels with significantly longer branches when compared to VAMPS generated with cyclopamine added to basal medium. These data suggest an interaction between these SHH, VEGF, and FGF2 signaling in VAMP models.

The BMPs, specifically BMP4, have been shown to be crucial for vascular tube formation in murine models.^63,64^ In VAMPs, addition of BMP4 between days 2 and 12 allowed for robust EC differentiation and vascular network formation [Fig. 7(h)]. Compared to controls, we detected no differences in vessel area, branch length, or vessel diameter when BMP4 was added [Fig. S5(b)]. The treatment with the BMP inhibitor LDN-193189 between days 2 and 12 produced VAMPs with a lower percentage of vessel area when compared to control VAMPs [Figs. 7(i) and S5(b)]. Furthermore, VAMPs generated with LDN-193189 treatment with basal medium had less vessel area compared to VAMPs generated with LDN-193189 combined with VEGF and FGF2. These findings align with previous findings from animal models.

Finally, we wanted to understand how arterial–venous identity was changing with differences in model conditions. We chose to further examine the effect of 8Br-cAMP on EC differentiation as we found the EC networks (or lack thereof) formed in the model well-represented the literature. We found, compared to the control, VAMPs treated with 8Br-cAMP resulted in vascular cells with increased levels of several arterial markers (*NOTCH4, GJA5,* and *EFBN2)* and decreased arterial marker *SOX17*. The venous marker *EPHB4* was also decreased, but we noted an increase in venous marker NRP2 (Fig. S4). This suggests there may be a change in arterial–venous identity within the model—in-depth analysis would be required to gain a more complete understanding.

## III. DISCUSSION

Human vascular development is a complex process that when impaired can lead to vascular malformations. A deep understanding of the key cellular and molecular events governing vascular development has been gained by animal model studies. The advent of human PSCs that can be differentiated into ECs and related vascular cells now enables us to study these events in humans. To enable such studies, we have established a new model, termed VAMP, that recapitulates vasculogenesis, angiogenesis, and arterial–venous EC fate divergence. Furthermore, we demonstrate the utility of the VAMP model by testing hypotheses regarding vascular development established in animal models and identify both similarities and differences that may point to conserved and unique features of human vascular development.

Isolated primary or 2D differentiated ECs have been used to assemble 3D vascular models. Some studies have reported that 2D differentiated hPSC-ECs can form a perfusable vascular network when cultured together with primary pericytes or fibroblasts,^36,65^ while others have reported that hPSC-ECs exhibited a fivefold reduction in capillary network formation compared to HUVECs.^66^ The experimental results are likely highly dependent on the EC stage. ECs change phenotype after isolation and expansion in 2D cultures, including losing arterial or venous characteristics; hence, the stage of EC culture is an important parameter when comparing protocols for network formation. In our case, 2D differentiation of human PSCs produced a mixture of ECs and vascular cells that was harvested on day 5 and immediately seeded into a 3D hydrogel within a microfluidic device. This approach successfully generated a perfusable vascular network with open lumen structures. Nevertheless, this assembled model did not demonstrate arterial–venous vessel specification.

During development, arterial or venous progenitors are believed to be generated from high and low VEGF environments, respectively. In hPSC differentiations, varying VEGF levels has been successfully implemented in order to promote arterial or venous fates.^3,4^ These directed differentiation approaches were designed to generate a single EC phenotype, but to study vascular development, we need a system that allows the maturation of both arterial and venous fates within the same culture. To achieve this, one approach is to produce a construct that includes different VEGF levels. However, growth factor concentration gradients are challenging to accomplish in a 2D culture. In VAMPs, we are able to capture diverging arterial and venous phenotypes and also demonstrate that these cells emerged from distinct locations within the model. We hypothesize that the way VAMPs are constructed created a VEGF gradient: high exogenous VEGF levels are present in the medium surrounding the encapsulated vascular spheroids, while lower levels of VEGF exist within the center of the vascular spheroid. We suggest this gradient is a key feature of VAMPs and is responsible for the localized venous-like ECs originating within the spheroid and for the observation of angiogenic ECs sprouting out from the spheroids, a known EC behavior that occurs in response to a VEGF gradient.^9^

Endothelial cell and mural cell crosstalk is crucial for regulating vasculogenesis, angiogenesis, and vessel maturation.^67^ Key ligands involved in EC–mural cell crosstalk include VEGF, TGFβ, PDGFβ, angiopoietins, sphingosine-1-phosphate, cadherin, and Notch. EC–mural cell interactions also play a vital role in vessel stability via vascular basement membrane deposition. The co-differentiation of EC and mural cells within a 3D environment enables VAMPs to mimic this multi-cellular crosstalk from the earliest stages. Dysfunction between the EC–mural cell relationship is observed in both congenital and acquired diseases, and therapies are being developed targeting these EC mural cell interactions. VAMPs are a viable human model system to screen candidate therapeutics that target this relationship.

Using VAMP constructs, we have evaluated the effect of various agonists/antagonists of several important signaling pathways in vascular development that have been established mostly from zebrafish or mouse models.^68,69^ We show that VAMPs respond to several key developmental signals identified in animal models, extending these findings to human cells. VAMPs responded to cAMP, Notch inhibition, and BMP as we predicted based on previous *in vivo* and *in vitro* studies. However, we found that VAMPs responded differently to certain activators/inhibitors depending on whether constructs were generated in the pro-angiogenic or in the pro-vasculogenic basal conditions. The most striking differential response was to treatments of the Notch inhibitor DAPT between days 2 and 12. In the presence of DAPT, vessel formation was inhibited in VAMPs generated in pro-angiogenic conditions with added VEGF and FGF2 but was apparently unperturbed in VAMPs generated in pro-vasculogenic basal conditions. Notch knockdown in mice has demonstrated global lack of angiogenic vascular remodeling, while early vasculogenesis appeared unaffected.^18^ Our data confirm this finding in a human model and show utility of VAMPs to study and modulate angiogenic vs vasculogenic vessel formation behaviors.

We have also identified treatments that produced effects dissimilar to those predicted from animal models. Most notably, from work performed in zebrafish, SHH is predicted to promote vascular differentiation and, being upstream of VEGF, to promote the arterial fate.^62,70^ SHH has also been identified as a pro-angiogenic signal through studies of mouse embryoid body models.^71^ In contrast, we found that treatment with the SHH agonist purmorphamine between days 2 and 12 potently inhibited human EC differentiation in VAMPs generated with or without additional VEGF and FGF2. Additionally, we found VAMPs treated with the SHH antagonist cyclopamine were still capable of forming vessel structures, albeit with some morphological differences. This may reflect a human-specific difference in the influence of SHH on vascular development. Another possible explanation for the discrepancy is a difference in morphogen presentation: normally, *in vivo*, SHH is secreted by structures such as the notochord and acts through local concentration gradients, which may not be captured when adding a soluble SHH mimic to an *in vitro* model.

In conclusion, in this work, we describe a protocol to efficiently and rapidly co-differentiate vascular ECs and mural supporting cells in 3D (VAMPs). VAMPs provide unique opportunities to investigate the human vasculature and have several advantages over previously published hPSC-derived 3D vascular models:^36,40,72,73^ (1) The protocol is serum-free and relies on minimal medium additives, which are attractive features for constructing robust, economical, and scalable *in vitro* vascular screens. (2) The protocol generates multiple vascular cell types with a single, simple protocol. Previous methods have mostly used 2D differentiated cells that were subsequently mixed together to produce a 3D structure. To form a robust functional vascular network, pericytes are needed, and in the cell mixing approach, pericytes are separately differentiated from hPSCs, which complicates the protocol and takes additional time. Co-deriving multiple cell types in 3D enables EC–mural cell interactions as these cells develop and promotes self-organization. This is a promising strategy for generating the required vascular cell types, arterial–venous-specification, and stable structures, and to enable studies of early aspects of vascular development. (3) VAMPs demonstrate evidence of early arterial–venous specification. Although these arterial-like and venous-like phenotypes do not fully recapitulate adult arterial or venous endothelial signatures, capturing evidence of early EC arterial–venous diversity is significant. This allows the investigation of arterial and venous cell interactions during the formation of blood vessels. Hence, VAMPs can be used as a platform to model various developmental vascular malformations due to genetic defects or environmental perturbations.

## IV. METHODS

### A. Cell culture and line information

hPSCs were maintained on Matrigel-coated dishes in mTESR1(StemCell Technologies catalog #85850), mTESR1 Plus (StemCell Technologies catalog #100-0276), or mTESR1 with FGF2-DISCs (StemCultures LLC, catalog # DSC500). HUVECs (Lonza) were cultured on 0.2% gelatin coated tissue culture dishes in EGM-2 medium (PromoCell catalog #C-22111). HUVECs were used at passage 4 or 5. The hESC line used in this work was WMC-2 carrying a VE-cadherin-promotor-mOrange reporter.^46^ hiPSC lines used in this work to validate finding in different cell lines from various donors were as follows: TCW1E33–1C6,^74^ TCW1E44-b,^74^ TCW2E33–2E3,^74^ TCW2E44–4B4,^74^ TCW3E33-RC1H,^74^ TCW4E33-MC2C,^74^ TCW4E44-RC2C,^74^ GIH-161,^75^ F11350,^75^ F12442,^76^ F11430,^76^ F12436,^76^ F12424,^76^ and CO0002.^77^

### B. 2D vascular differentiation

When hPSCs reached approximately 80% confluency, hPSCs were single cell-passaged using Accutase (Gibco catalog #A1110501) and replated at cell density of 35 000 or 50 000 cells/cm^2^ on Matrigel-coated tissue culture plates. hPSCs were cultured overnight in PSC medium supplemented with 10 *μ*M Y-27632 (Tocris catalog #1254). If PSCs were seeded at 35 000 cells/cm^2^, hPSCs were cultured for another 1–2 days prior to starting the differentiation. If PSCs were seeded at 50 000 cells/cm^2^, differentiation was started the following day. To start the differentiation, hPSC medium was replaced with LaSR medium comprised of Advanced DMEM/F12 (Gibco catalog #12634010), GlutaMAX (Gibco catalog #35050061), and 60 *μ*g/ml of L-ascorbic acid (Sigma catalog #A-4403 or Sigma catalog #A-8960) and supplemented with 6 *μ*M of CHIR99021 (Tocris catalog #4423) and cultured for 2 days to pattern cells toward mesoderm. On day 2, the medium was fully removed and replaced with LaSR medium supplemented with 50 ng/ml VEGF_165_ (Peprotech catalog #100–20, Shenandoah catalog #100-44 or R&D catalog #293-VEGF) and 20 ng/ml FGF2 (Peprotech catalog #AF-100-18B or Shenandoah Biotechnology catalog #100-28), unless otherwise stated. ECs were sorted between days 5 and 8 using either VE-cadherin (Miltenyi Biotec catalog #130-097-857) or CD31 magnetic beads (Miltenyi Biotec catalog #130-091-935), or FACs sorted with CD31 antibody (BD Biosciences catalog #550389 or Invitrogen catalog #17-0319-41). Sorted hPSC-ECs were plated at a density between 50 000 and 100 000 cells/cm^2^, and cultured on 0.2% gelatin-coated or Matrigel-coated dishes in either EGM-2 medium (PromoCell catalog #C-39211) or LaSR medium supplemented with 10% FBS, 25 ng/ml VEGF, and 20 ng/ml FGF2.

### C. Encapsulation of hPSC spheroids for VAMPs

To form hPSC spheroids, hPSCs were dissociated into single cells using Accutase and seeded into AggreWell plates (StemCell Technologies catalog #34425) at a density of about 250 000–500 000 cells per well in mTESR1 or mTESR1Plus supplemented with 10 *μ*M Y-27632 (Tocris catalog #1254) and cultured overnight. The following day, spheroids were encapsulated into fibrin gels. First, fibrinogen solution was made fresh, dissolving fibrinogen powder (Sigma catalog #F8630–5G) in DPBS^++^ at 20 mg/ml and syringe filtered through a 0.22 *μ*m membrane for sterility. Then spheroids were removed from AggreWell plates by pipetting up and down with a 1000 *μ*l pipette tip. Spheroids generated from one AggreWell were transferred to a conical tube, washed twice in PBS and resuspended in about 300 *μ*l of LaSR medium. Thrombin (Sigma catalog #T4648–1KU or catalog #T9549), stored frozen in aliquots, was added to spheroid suspension at a concentration of 4–8 units/ml. Aprotinin (Fisher catalog #BP250310) was also added to the spheroid suspension solution at a concentration of 10 *μ*g/ml. Next, Eppendorf tubes were loaded with 25 *μ*l of well mixed spheroid suspension, for 12 tubes in total. To create gels, 25 *μ*l of fibrinogen solution was added to one Eppendorf tube, mixed thoroughly, and two 25 *μ*l droplets were quickly pipetted into the middle of a well from a 12- or 24-well plate. This was repeated using the remaining reaction tubes, thus creating 24 models from one well of an AggreWell plate. Final concentrations within each model are 10 mg/ml of fibrinogen, 2–4 units/ml of thrombin, 5 *μ*g/ml (30 KIU) of aprotinin, and approximately 10–30 spheroids. Fibrin gels were allowed to polymerize at room temperature for about 30 min prior to adding medium to each well.

### D. 3D vascular differentiation for VAMPs

After encapsulation of PSC spheroids into fibrin gels, differentiation was begun by adding LaSR medium supplemented with 6 *μ*M of CHIR99021 and 5 *μ*g/ml (30 KIU) of aprotinin. On day 2, a full medium exchange was performed and LaSR medium supplemented with 25–50 ng/ml VEGF and 20 ng/ml FGF2 was added (no aprotinin), except in basal conditions in which no VEGF or FGF2 was added. On day 4, 50% medium exchange was performed to include 5 *μ*g/ml of aprotinin and continued every 3–4 days. For VAMPs generated for developmental signal screens (see Fig. 6), VAMPs were treated every 3 days with additional small molecules or growth factors between day 2 and day 12 during medium changes at the following final concentrations: 20 *μ*M DAPT (Tocris catalog #2634), 100 *μ*M 8Br-cAMP (Tocris catalog #1140), 20 ng/ml BMP4 (Peprotech, catalog #120-05SET), 250 nM LDN-193189 (Tocris, catalog #6053), 6 *μ*M CHIR99021 (Tocris, catalog #4423), 5 *μ*M IWP2 (Tocris catalog #3533), 2 *μ*M purmorphamine (Tocris, catalog #4551), and 2 *μ*M cyclopamine (Tocris, catalog #1623).

### E. Single cell dissociation of VAMPs

Collagenase solution was freshly made from collagenase powder (Sigma) dissolved in 5 mM PBS (with calcium and magnesium ions), 20% FBS, and sterile water to a final concentration of 2 mg/ml. Fibrin gels were exposed to the collagenase solution for 1 h on a shaker at 37 °C in a sterile incubator. The gels were pipetted constantly for under 2 min to release cells and ECM using a micropipette. After washing in DMEM/F12 and re-suspended in Dispase (STEMCELL Technologies), the solution was once again placed in the sterile incubator on a shaker at 37 °C for 20 min. The resulting cell clumps were washed in DMEM/F12 and resuspended in Accutase (Gibco) for 20 min under the same incubating conditions. The cells were pipetted constantly for under 2 min and, after examination under the microscope to ensure a single cell population, washed with PBS (with no calcium and magnesium).

### F. Encapsulation and differentiation of hPSC spheroids into VAMPs within a microfluidic device

To form hPSC spheroids, hPSCs were dissociated into single cells using Accutase and seeded into AggreWell plates (StemCell Technologies catalog #34425) at a density of about 250 000–500 000 cells per well in mTESR1 or mTESR1Plus supplemented with 10 *μ*M Y-27632 (Tocris catalog #1254) and cultured overnight. The following day, spheroids were encapsulated into fibrin: to encapsulate cells into the hydrogel, 10 *μ*l of cell suspension in fibrinogen and 10 *μ*l of thrombin solution (4 units/ml PBS^++^) were thoroughly mixed and immediately pipetted into the hydrogel channel of the microfluidic device (AIM Biotech catalog #DAX-1) and allowed to polymerize at 37 °C and 5% CO_2_ for 15 min. The resulting fibrin gel was comprised of 4 mg/ml of fibrinogen, 2 unit/ml of thrombin, and 20–30 spheroids/microfluidic device.

hPSC-EC differentiation was performed following the method described above but using 100 ng/ml of VEGF (Peprotech catalog #100–20) and 20 ng/ml FGF2 (Peprotech catalog #AF-100-18B) between days 2 and 10. To induce interstitial flow across the fibrin gel, 80 *μ*l of the medium was added to both reservoirs on one side of the hydrogel channel and 40 *μ*l was added to both reservoirs on the opposite side. The medium was replenished daily to re-establish reservoir volumes and maintain the hydrostatic pressure gradient across the hydrogel channel.

### G. hPSC-derived 2D vascular cells seeded into microfluidic model for 3D vascular model

hPSC-EC differentiation was performed following the method described above but using 100 ng/ml of VEGF (Peprotech catalog #100-20) and 20 ng/ml FGF2 (Peprotech catalog #AF-100-18B) between days 2 and 6. On day 6, differentiated vascular cells were harvested using Accutase and resuspended at 2 × 10^7^ cells/ml in freshly prepared solution of 10 mg/ml fibrinogen dissolved in EGM-2 medium. To encapsulate cells into the hydrogel, 10 *μ*l of cell suspension in fibrinogen and 10 *μ*l of thrombin solution (4 units/ml PBS^++^) were thoroughly mixed, immediately pipetted into the hydrogel channel of the microfluidic device (AIM Biotech catalog #DAX-1) and allowed to polymerize at 37 °C and 5% CO_2_ for 15 min. The resulting fibrin gel was comprised of 5 mg/ml of fibrinogen, 2 unit/ml of thrombin, and 1 × 10^7^ cells/ml. After fibrin polymerization, EGM-2 supplemented with 5 *μ*g/ml of aprotinin and 50 ng/ml of VEGF was introduced to the four microfluidic device reservoirs. To induce interstitial flow across the fibrin gel, 80 *μ*l of the medium was added to both reservoirs on one side of the hydrogel channel and 40 *μ*l was added to both reservoirs on the opposite side. The medium was replenished daily to re-establish reservoir volumes and maintain the hydrostatic pressure gradient across the hydrogel channel. After 3 days of culture within the microfluidic device, sorted hPSC-ECs were seeded on either side of the hydrogel channel to help promote anastomosis. hPSC-ECs were resuspended at a concentration of 2 × 10^6^ cells/ml in EGM-2 medium, added into one media channel and incubated at 37 °C and 5% CO_2_ with the device positioned sideways (rotated 90 degrees) for 30 minutes to allow cells to adhere along the hydrogel channel. Afterward, the non-adherent cells were cleared out of the device by washing with EGM-2, and the process was repeated for the opposite media channel.

### H. Microfluidic microsphere perfusion assay

To confirm the perfusion of microvessels formed in microfluidic devices, a solution of Dragon Green^TM^ (Bangs Laboratories catalog #FSDG005) uniform dyed polystyrene microspheres (∼2 *μ*m diameter) was diluted in EGM-2 at a ratio of 1:1000. Next, 40 *μ*l of unmodified EGM-2 was added to both reservoirs of one microfluidic channel and 50 *μ*l of microbead solution was added to both reservoirs of the opposite channel. Immediately afterward, fluorescence time lapse images were acquired for 30 s at 200 ms intervals with an Eclipse Ti2 Microscope. Maximum intensity projection images were created to visualize the paths of the microspheres through the open lumen of the microvessels.

### I. Microvessel image analysis

To quantify the effects of development cues on vessel structures, an image analysis was performed. The analysis was carried out as previously shown.^78^ Fluorescence images were acquired using a Nikon Eclipse Ti2 fluorescent microscope with a 10× objective. The VPr-mOrange/VE-cadherin reporter was used to detect the microvascular networks. The fluorescent signal was quantified and used to calculate blood vessel parameters (blood vessel area, average branch length, and average branch diameter) for all experimental conditions. Using ImageJ, the fluorescent signal was converted to a binary image and thresholding was carried out to subtract individual particles smaller than an individual endothelial cell, paying particular attention to the removal of background fluorescence and out of focus structures. The vessel area (AV) and percentage of image containing vessels was calculated from the thresholded image. “Skeletonize” was used to outline the framework of the vessel network and “Analyze Skeleton (2D/3D)” was used to determine the number of branches (nB) and the average branch length (LB). The identical equation as used previously was applied to calculate the average branch diameter (DB),

$$D_{B}=\frac{A_{V}}{n_{B}\times L_{B}}.$$

For all generated blood vessel parameters, 3–6 experimental replicates were analyzed and up to three ROI in each replicate were studied.

### J. Statistics

Data are presented as a mean and standard error. For comparisons between two groups, unpaired two-tailed student t-tests were performed, and p-values ≤ 0.05 was considered statistically significant. For comparisons between three or more groups, one-way ANOVA, post hoc Dunnett's test with control sample set to “HUVECs” and p-values ≤ 0.05 was considered statistically significant. Statistical analysis was performed using GraphPad Prism (La Jolla, CA).

## SUPPLEMENTARY MATERIAL

See the supplementary material for the description of figures, tables, methods, and videos.

## Data Availability

The data that support the findings of this study are available within the article and its supplementary material.
